# Clinical dietitian-led nutrition counseling and exercise to reduce cardiovascular risk in adults living with a BMI above 27 and severe mental illness: the NORMI-Heart trial protocol

**DOI:** 10.3389/fnut.2026.1700251

**Published:** 2026-02-12

**Authors:** Madeleine Elisabeth Angelsen, Hemen Najar, Mette Svendsen, Magne Thoresen, Dawn Elizabeth Peleikis, Kjetil Retterstøl

**Affiliations:** 1 Department of Nutrition, Faculty of Medicine, Institute of Basic Medical Sciences, University of Oslo, Oslo, Norway; 2 Department of Psychotic Disorders, Sahlgrenska University Hospital, Västra Götaland Region, Gothenburg, Sweden; 3 Institute of Health and Care Sciences, University of Gothenburg, Gothenburg, Sweden; 4 Section for Preventive Cardiology, Department for Endocrinology, Obesity and Preventive Medicine, Oslo University Hospital, Oslo, Norway; 5 Department of Biostatistics, Faculty of Medicine, Institute of Basic Medical Sciences, University of Oslo, Oslo, Norway; 6 District Psychiatric Center (DPS) of Asker, Vestre Viken Hospital Trust, Drammen, Norway; 7 The Lipid Clinic, Medical Department, Aker Hospital (Aker Sykehus), Oslo University Hospital (Oslo universitetssykehus), Oslo, Norway

**Keywords:** cardiovascular disease, clinical dietitian, heart health, lifestyle intervention, metabolic risk factor, psychiatry, severe mental illness, weight reduction

## Abstract

**Background:**

Severe mental illness (SMI) is associated with high cardiovascular disease (CVD) risk. Important contributors are poor diet quality, physical inactivity, and metabolic side effects of antipsychotic medication entailing significant weight gain. Evidence implies that dietary counseling delivered by clinical dietitians leads to greater improvements in diet quality, weight management, and cardiometabolic health than counseling delivered by non-specialist health personnel, especially when combined with increased physical activity. However, randomized controlled trials (RCTs) testing clinical dietitian-led nutrition counseling integrated with structured exercise across diverse treatment modalities in psychiatric care remain scarce.

**Objective:**

The Norwegian Mental Illness Heart Health (NORMI-Heart) study will evaluate the efficacy and applicability of a 6-month lifestyle program, combining individualized, clinical dietitian-led nutrition counseling focused on weight reduction and improved dietary quality, and moderate-to-vigorous exercise training, on CVD risk in adults with SMI in Oslo, Norway.

**Methods:**

This parallel-group RCT will recruit 70 overweight adults with SMI. Participants will be randomized to a 6-month diet and lifestyle intervention, or control. The intervention is individualized and includes monthly one-to-one sessions with a clinical dietitian focusing on heart-healthy eating and weight reduction, monthly supervised group exercise, and support to follow an individual exercise program. Control participants will receive treatment as usual (TAU). The NORMI-Heart Trial aims to evaluate the effect of the intervention on estimated CVD risk.

**Expected outcomes:**

This trial aims to provide evidence on the effectiveness, feasibility, and real-world applicability of clinical dietitian-led nutrition counseling combined with structured exercise for reducing CVD risk in individuals with SMI. Findings may strengthen the scientific and policy rationale for integrating clinical dietitians as a standard part of multidisciplinary mental health care. Results will also inform scalable, integrated lifestyle care models in mental health services.

**Trial registration:**

ClinicalTrials.gov Identifier: [NCT07085923].

## 1 Introduction

Schizophrenia and bipolar disorder are severe mental illnesses (SMIs) that significantly impair quality of life and functional ability, often debuting in early adulthood and requiring long-term treatment with psychotropic medication like antipsychotics (1, 2). Many antipsychotics are associated with adverse metabolic side effects such as weight gain, dyslipidaemia, and hyperglycaemia (3), contributing to the development of the metabolic syndrome (4), which in turn increases the risk for cardiovascular diseases (CVDs) (5).

Individuals with SMI have a life expectancy approximately 10–20 years shorter than the general population, primarily due to natural causes like CVDs (6, 7). Despite major advances in prevention and treatment of CVDs in recent decades, people living with SMI have not experienced the same decline in CVD mortality as the general population (8). This excess mortality is also evident in Norwegian cohorts and cannot be fully explained by lower socioeconomic status or educational attainment (9).

In addition to increased CVD mortality, individuals with SMI have an elevated risk of cognitive impairment and dementia compared with the general population (10). Dementia and CVD share many overlapping risk factors, including metabolic dysregulation, inflammation, and vascular pathology (11, 12). Dietary components have also been shown to influence trajectories of neurocognitive decline (13). Plasma biomarkers such as phosphorylated tau-217 (p-tau217) and neurofilament light chain (NfL) are indicators of neurocognitive decline predictive of dementia and Alzheimer's disease (14, 15), and recent findings suggest elevations of NfL among individuals with SMI (16).

Scandinavian studies consistently report high rates of obesity and metabolic risk factors for CVD among individuals with SMI (17, 18). Similar findings were documented in recent master's projects conducted in a municipal psychiatric treatment unit in Norway (19–21), revealing unhealthy dietary habits, low levels of physical activity, and high rates of smoking. These findings represent modifiable CVD risk factors that can be targeted through lifestyle interventions.

In Denmark, patients with SMI were found to receive less medical treatment for CVD risk factors, including diabetes, compared with the general population (22). This is further confirmed in international studies that have reported lower levels of somatic care for outpatients with SMI (23). In Norway, national guidelines recommend integration of somatic screening and lifestyle education in psychiatric care (24). Despite this, clinical experiences suggests that implementation remains limited in practice (25).

CVD-risk can be estimated using validated tools that combine multiple risk enhancers to predict the likelihood of a cardiovascular event within a given time frame. QRISK3 (CVD Risk Algorithm, version 3) is one such tool, developed to estimate an individual's 10-year risk of heart attack or stroke, and notably includes SMI and antipsychotic medication as independent risk factors (26). Previous studies have shown that lifestyle interventions promoting healthy weight, dietary improvements and increased physical activity can reduce CVD-risk estimates by modifying underlying risk enhancers (27, 28). However, such reductions in predicted CVD risk have not been demonstrated in SMI populations, where both adherence to lifestyle measures and baseline risk profiles may differ.

Previous research on lifestyle interventions for individuals with SMI has produced heterogeneous results, which may be due to different study settings and -design as well as methodological limitations. While early trials, such as the Finnish Mental Hospital studies, suggested a strong protective effect of dietary fat modification on cardiovascular outcomes in inpatients with SMI (29, 30), more recent findings on diet- and healthy lifestyle interventions remain mixed. Several systematic reviews highlight modest effects on weight and metabolic outcomes (31, 32), while others emphasize limited clinical relevance and substantial methodological variability (33, 34). Importantly, conflicting results regarding efficacy of lifestyle interventions highlight that nutrition counseling delivered by clinical dietitians is more effective than dietary interventions conducted by non-specialists (31, 34, 35). Another key factor for achieving weight loss and reducing cardiometabolic risk parameters in individuals with SMI is the incorporation of moderate- to vigorous-intensity physical activity alongside dietary improvements (31). Further, peer-specialist support and a maintained user perspective may be an important factor for successful implementation and long-term adherence to lifestyle changes (36).

### 1.1 Rationale for the present study

The present study is a randomized controlled trial (RCT) designed to test whether a lifestyle intervention can reduce CVD risk in individuals with a body mass index (BMI) above 27 kg/m^2^ and SMI. Recent European guidelines underscore the importance of integrating lifestyle interventions into routine psychiatric care for this population (37). In line with these recommendations, the intervention tested in this RCT combines the two most consistently reported success factors in the literature: application of moderate-to-vigorous physical activity (31) and nutrition counseling led by clinical dietitians (32, 35).

Drawing on the research team's clinical and research related experience with individuals living with SMI, the intervention is adapted to be feasible and scalable across diverse psychiatric treatment settings. Particular attention is paid to aligning the intervention with the real-world constraints and therapeutic contexts commonly encountered in this population. Furthermore, input from a user panel with lived experiences has informed the final design, especially highlighting the potential value of involving significant others (S.O.s) to support adherence and long-term outcomes. Additionally, the Goal-Based Outcomes (GBO) tool, a patient-centered process measure validated in mental health services, will be incorporated to capture participants' self-defined goals and perceived progress across domains of the intervention (38) (Appendices).

Including biomarkers of neurocognitive decline (NfL and ptau-217) in exploratory analyses may further shed light on the relationship between neurocognitive and cardiometabolic vulnerability in SMI and enable examination of how biomarkers of these pathologies co-vary throughout the study period. Supplementation with B-vitamins have shown potential to improve biomarkers of cognitive decline when omega-3 status is adequate (39).

### 1.2 Aim, research question, hypothesis, and objectives

#### 1.2.1 Study aim

To evaluate the effect of a 6-month lifestyle intervention on estimated CVD risk in individuals with a BMI ≥27 kg/m^2^ who receive treatment for SMI across several psychiatric care settings in the Norwegian capital.

#### 1.2.2 Primary research question

Does participation in a structured lifestyle intervention lead to a change in estimated 10-year CVD risk, as measured by QRISK3, compared with TAU in individuals with a BMI ≥27 kg/m^2^ and SMI?

#### 1.2.3 Primary hypothesis

There will be a difference in the change in QRISK3 scores from baseline to 6 months between the intervention and control groups.

#### 1.2.4 Secondary hypothesis

Improvements in adherence to a healthy lifestyle, measured by the DIGIKOST (Digital Dietary Questionnaire) Lifestyle Index, a questionnaire-based composite of diet, physical activity, tobacco, and alcohol habits (40), will be associated with reductions in QRISK3 scores over the intervention period.

## 2 Methods and analysis

### 2.1 Study design and setting

The **Nor**wegian **M**ental **I**llness **Heart** Health (NORMI-Heart) study is a parallel-group, RCT with a 1:1 allocation ratio, conducted across different psychiatric care settings in Oslo, the capitol of Norway.

#### 2.1.1 Study design

**Figure 1:**
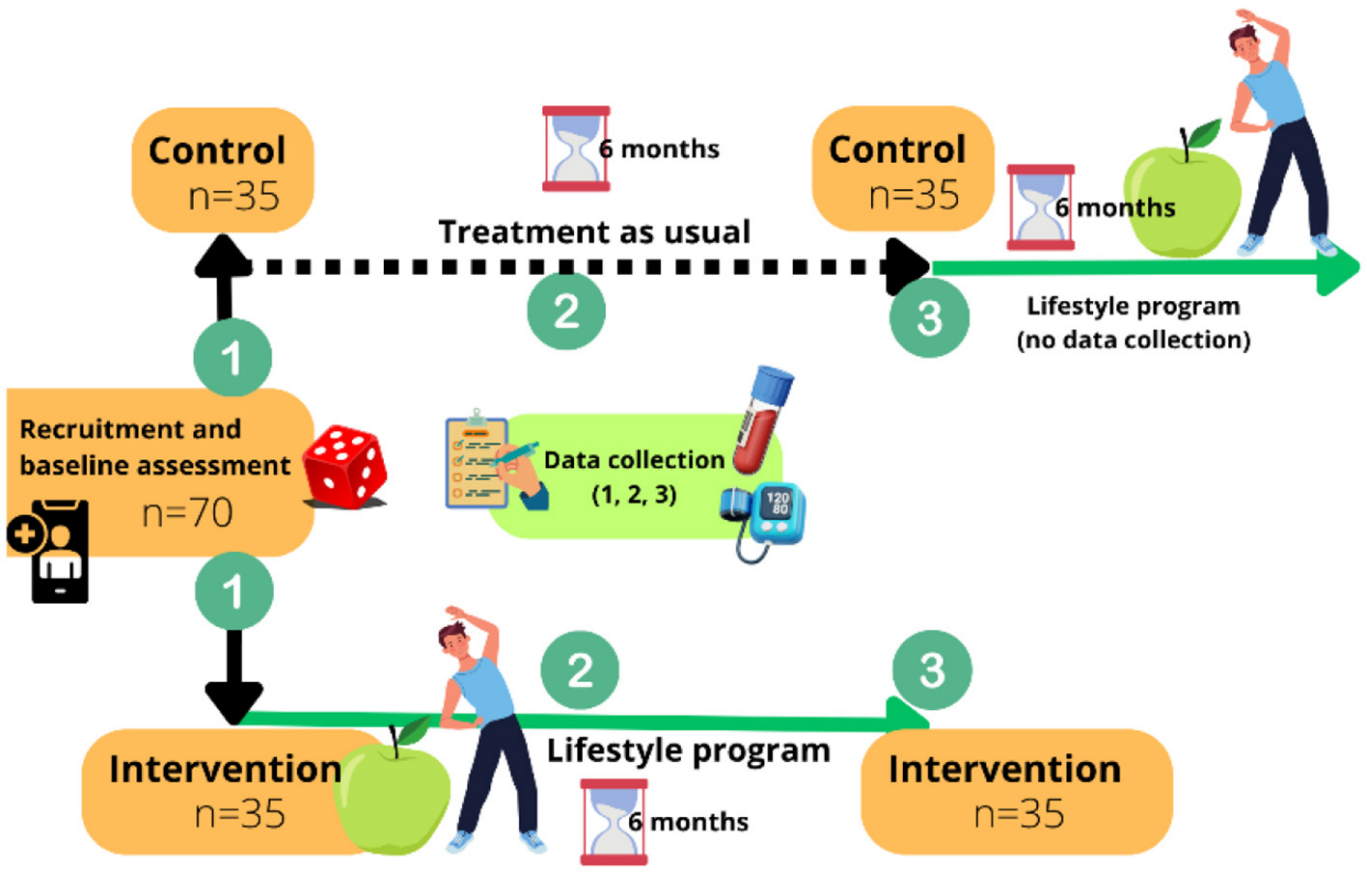
Overview of trial design. Participants randomized to the intervention group takes part in a combined lifestyle program for 6 months, while the control group receives treatment as usual. Control participants are offered the same lifestyle program as the intervention group after data collection.

A total of 70 participants will be randomized to either the intervention group, receiving a structured lifestyle program in addition to treatment as usual (TAU), or the control group, receiving TAU alone for 6 months (Figure 1). The trial follows a superiority framework and is designed to test whether a combined diet and physical activity intervention reduces CVD risk more effectively than TAU alone among adults receiving treatment for SMI across various psychiatric care settings in Norway.

The intervention period is 6 months, with outcome assessments conducted in both arms at baseline (0 months), mid-intervention (3 months), and post-intervention (6 months). After data collection is finalized, control group participants will be offered the same lifestyle program to ensure ethical balance, however, no further data will be collected during this phase. The study is open-label due to the nature of the intervention. A visual overview of the trial flow is presented in Figure 1.

#### 2.1.2 Study setting

The trial is conducted in Oslo, the capitol of Norway, and reflects real-world psychiatric care by including a range of treatment contexts. Eligible participants are recruited from specialist mental health services, including:

- Flexible Assertive Community Treatment (FACT) teams.
- District Psychiatric Centers.
- Specialized outpatient clinics.
- Twenty four hour inpatient psychiatric units.

These services are affiliated with public and private non-profit hospitals in Oslo, including Lovisenberg Diaconal Hospital, Diakonhjemmet Hospital and Oslo University Hospital.

Baseline and follow-up assessments, dietary counseling sessions, and study coordination are conducted at Domus Medica, University of Oslo, a centrally located facility with good access from both treatment sites and surrounding residential areas in Oslo.

### 2.2 Participants and recruitment

#### 2.2.1 Eligibility criteria

Eligible participants are adults with a BMI exceeding overweight (≥27 kg/m^2^) and a verified SMI diagnosis, who are currently prescribed antipsychotic medication and/or lithium. SMI diagnoses are defined according to the International Classification of Diseases, 10th Revision (ICD-10), where codes F20.0–F29 include schizophrenia, schizotypal, schizoaffective, delusional, and other non-organic psychotic disorders, and codes F31.0–F31.9 represent bipolar affective disorder. Both bipolar I and II disorders are included, as individuals meeting the remaining eligibility criteria are expected to exhibit a comparable degree of psychiatric symptom burden and functional impairment, consistent with evidence on the long-term diagnostic stability and clinical overlap of bipolar affective disorders (41). Eligible participants must have a BMI exceeding 27 kg/m^2^, corresponding to overweight or obesity. This threshold was chosen to include individuals with a higher likelihood of cardiometabolic vulnerability and potential for clinically meaningful health benefits from weight reduction, consistent with international criteria for pharmacological weight management (42). All participants must be able to provide informed consent. A complete overview of inclusion and exclusion criteria is presented in Table 1.

**Table 1:** Eligibility and Exclusion criteria for participation.

**Inclusion criteria**	**Exclusion criteria**
Age 25–69 years	• Inability to provide informed consent • Significant cognitive impairment^1^ • Acute psychiatric crisis^a^
**Diagnosis of (ICD-10)**^**a**^ • Schizophrenia Spectrum (F20-29) • Bipolar Affective Disorder (F31.0-9)	**Initiation of medication during trial period**^**b**^ • GLP1-analouges • Lipid-lowering medication • Antidiabetics • Antihypertensives
BMI ≥27 kg/m^b^	**Clinical status** • Pregnancy • Type 1 diabetes • Established CVD • Medical inability to perform physical exercise
Prescription of antipsychotic agent and/or lithium	**High risk somatic findings** • HbA1c >57 mmol/L • LDL-C >5 mmol/L • Blood Pressure >180/100 mmHg • Malignant disease
Considered competent to consent^a^	**High alcohol intake** • >14 units/week for men • >7 units per week for women

^a^Diagnostic eligibility will be verified by the responsible treatment provider based on medical records. Diagnoses are classified according to the International Classification of Diseases, 10th Revision (ICD-10), where codes F20.0–F29 include schizophrenia, schizotypal, schizoaffective, delusional, and other non-organic psychotic disorders, and codes F31.0–F31.9 represent bipolar affective disorder. ^b^ Initiation of these medications during the study period will result in exclusion from per-protocol analyses, but participants will remain in the intention-to-treat population unless withdrawal is requested, or clinical discontinuation is deemed necessary. ICD; International Classification of Diseases, GLP1; Glucagon Like Peptide 1, BMI; Body Mass Index, CVD; Cardiovascular Disease, HbA1c; Glycosylated hemoglobin B, LDL-C; Low Density Lipoprotein Cholesterol.

#### 2.2.2 Recruitment procedures

**Figure 2:**
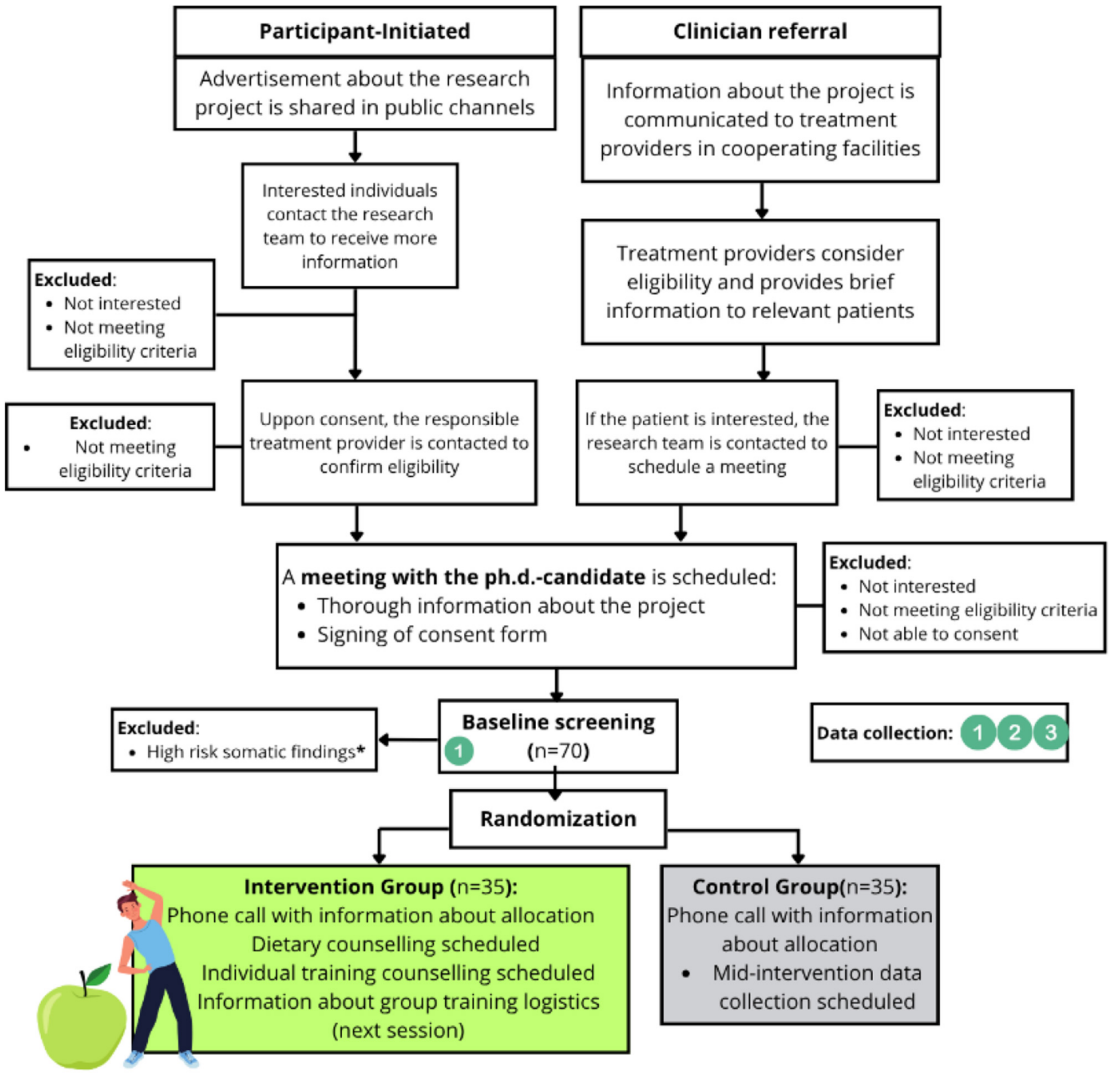
Flow Chart of Recruitment Pathway. Cooperating facilities in Oslo include Lovisenberg Diaconal Hospital, Oslo University Hospital and Diakonhjemmet Hospital, covering all treatment modalities. Baseline screening is conducted prior to randomization. *High risk somatic findings indicating pharmaceutical intervention is listed in Table 1.

Participants will be recruited via two complementary pathways: (1) referral by mental health professionals at collaborating institutions (Lovisenberg Diaconal Hospital, Oslo University Hospital, Diakonhjemmet Hospital), or (2) self-referral following public announcements or outreach. Individuals with SMI not currently receiving care at the collaborating hospitals may also participate if their psychiatric treatment provider confirms eligibility and agrees to be involved. This dual approach ensures broad accessibility while maintaining clinical oversight. The recruitment pathway, including cooperating facilities in Oslo and baseline screening prior to randomization, is illustrated in Figure 2.

#### 2.2.3 Informed consent

Written Patient Information Sheet (PIS) (Appendices) will be obtained from all participants prior to any study-related procedures. Participants will receive detailed oral and written information about the study and will have the opportunity to ask questions and consider participation without feeling obliged to consent to their treatment provider. Translated versions of the consent documents will be made available if needed. The consent process has been approved by the Regional Committees for Medical and Health Research Ethics in Norway (reference: 865976).

#### 2.2.4 Randomization process

Participants who provide informed consent and meet the eligibility criteria will be randomized to either the intervention or control group in a 1:1 ratio. Randomization will be stratified by diagnostic group (schizophrenia spectrum- or bipolar affective disorder), using a simple randomization list with permuted blocks generated in advance by the project's biostatistician. The allocation sequence will be securely stored by the biostatistician, who is not involved in participant recruitment or data collection. Once a participant has completed baseline assessments, the biostatistician will receive the diagnostic category and disclose the assigned group. This procedure ensures allocation concealment until baseline data are collected. Due to the nature of the intervention, neither participants nor intervention providers will be blinded to group allocation.

#### 2.2.5 Blinding and bias mitigation

Outcome assessors are blinded to group allocation during all baseline assessments, as randomization takes place only after baseline data are collected. To minimize contamination in the control group, dietary assessments (DIGIKOST and 24-h recalls) are conducted only at baseline and 6 months. Assessors are trained to provide neutral instructions, and participants are encouraged to answer truthfully.

Objective outcomes follow standardized measurement procedures. Statistical analyses will be conducted by the PhD candidate with guidance from the project statistician. As the primary outcome is objectively estimated and baseline assessments are blinded, additional blinding during analysis is not required.

#### 2.2.6 Individual dietary counseling

**Figure 3:**
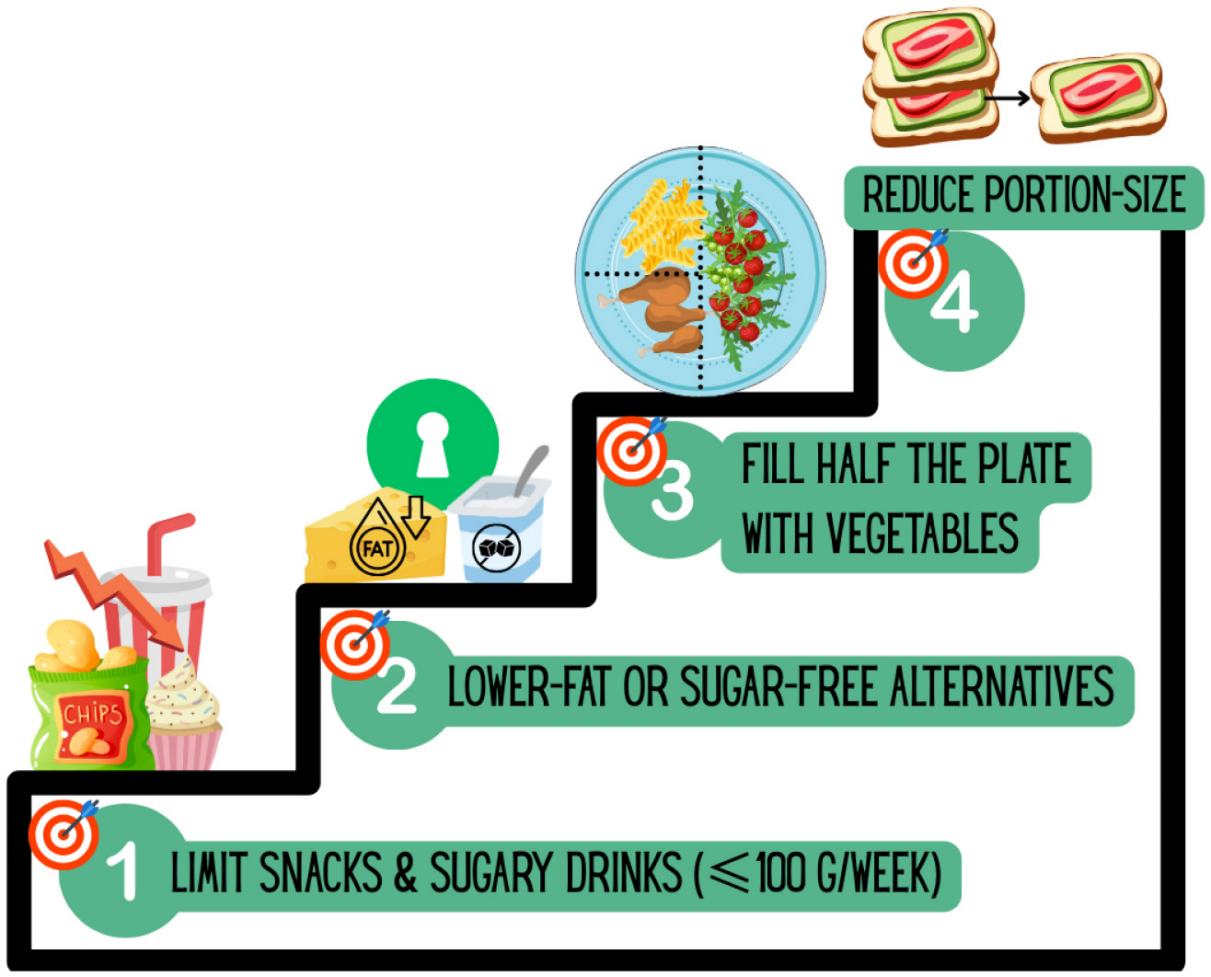
Four-step dietary framework from the LEVA trial. The LEVA intervention targeted sustainable energy reduction through sequential goals: (1) limit snacks and sugar-sweetened beverages to ≤100 g/week, (2) replace foods with lower-fat or sugar-free alternatives (e.g., Keyhole-labeled products), (3) fill half of the plate with vegetables, and (4) reduce portion sizes by equally reducing carbohydrate- and fat sources.

**Figure 4:**
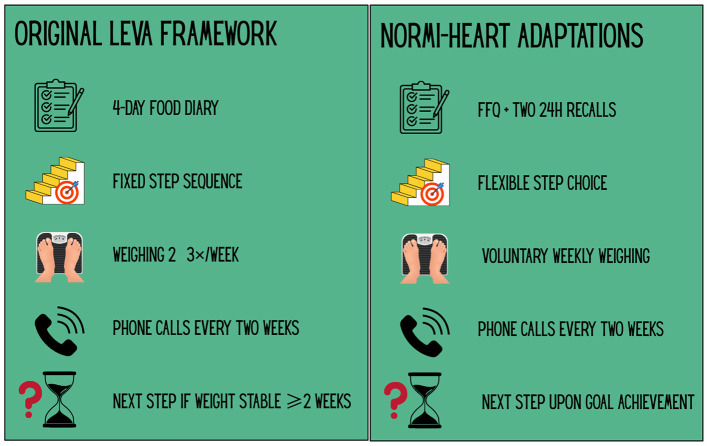
Comparison of the LEVA framework and the adapted approach. Adaptations made in the current trial, as opposed to the original LEVA framework, were designed to improve feasibility and acceptability for individuals with severe mental illness (SMI). Icons illustrate the corresponding elements in each framework: dietary assessment method, sequencing of dietary steps, weight monitoring routines, biweekly follow-up phone calls, and criteria for initiating the next dietary step.

Monthly counseling sessions are delivered by a registered clinical dietitian at Domus Medica, University of Oslo. The sessions follow a structured framework inspired by a previous Scandinavian study, the LEVA-trial, which demonstrated long-term effects on energy intake and diet quality in overweight and lactating women (43). In the present study, the dietary approach has been adapted to the needs and capacities of individuals with SMI, while maintaining its focus on stepwise behavioral change and sustainable caloric reduction. The four-step dietary framework from the LEVA trial is illustrated in Figure 3, while Figure 4 compares the original LEVA method with its adaptations in the NORMI-Heart trial. The process of adapting the LEVA framework was informed by input from a user advisory panel and a resource group of clinical dietitians experienced in both psychiatric- and obesity treatment.

In the current trial, participants are supported to select and implement one dietary step (either 1-4) at a time, guided by their preferences, readiness, and weight trajectory. Behavioral strategies include food logs to support reflection, recommended home weight monitoring 1–2 times per week (with project scales provided when needed), and biweekly follow-up phone calls to review weight development, adherence, and readiness to advance. If participants prefer not to weigh themselves at home, alternative monitoring methods such as waist measurement may be considered and discussed during follow-up calls. In each consultation, the GBO tool (38) (Appendices) is applied to capture participants' self-defined goals and perceived progress over time.

#### 2.2.7 Group-based physical activity sessions

Participants are invited to monthly supervised group training sessions led by qualified instructors affiliated with the Norwegian Sport Science Academy (Norges Idrettshøgskole, NIH). These sessions aim to:

- Support participants in achieving at least 150 min of moderate-to-vigorous physical activity per week.
- Focus on inclusive and accessible activities tailored to functional level, including walking, bodyweight circuits, ball games, and aerobic routines.
- Foster motivation, social support, and skill-building for implementing activity between sessions.

### 2.3 Intervention

**Figure 5:**
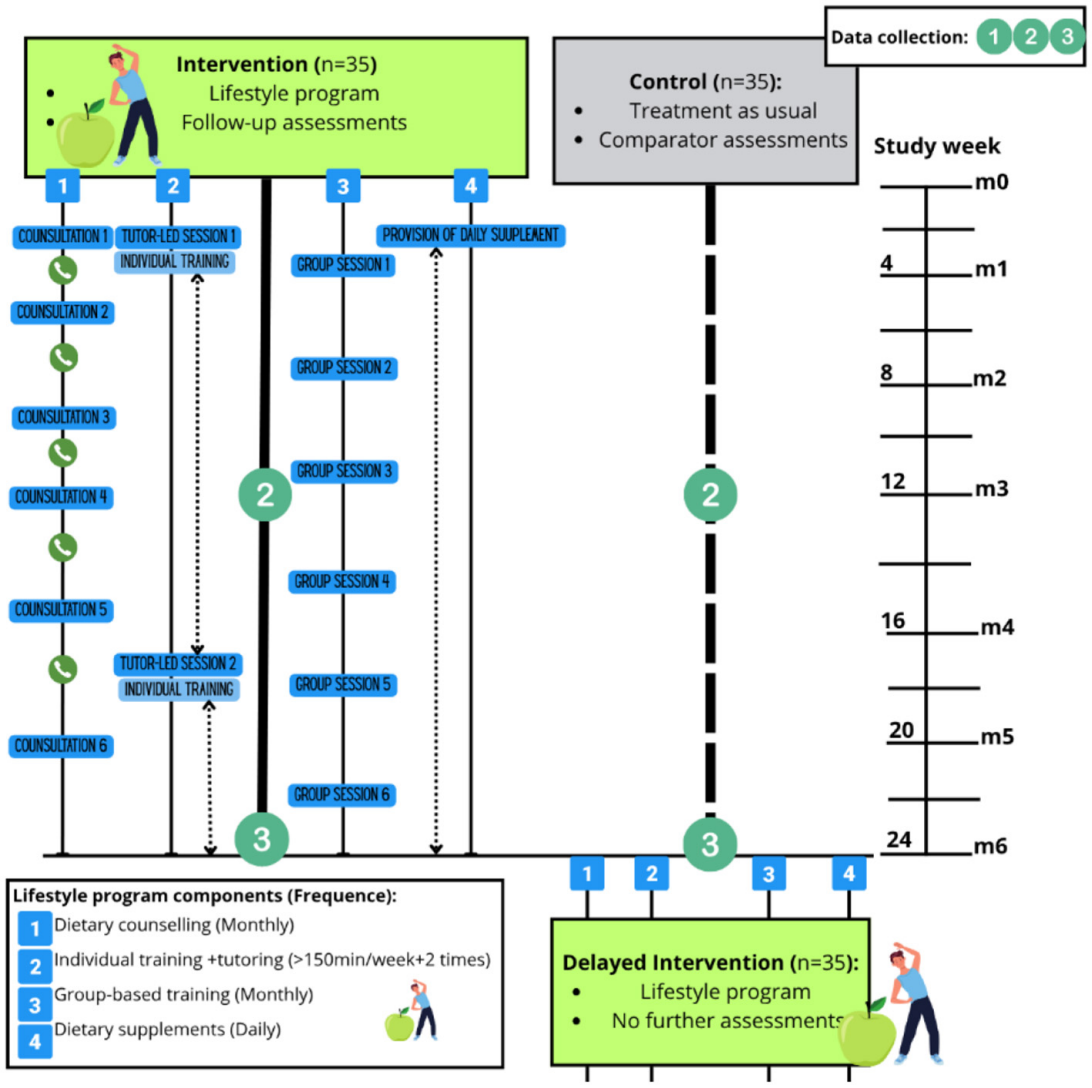
Intervention components, TAU, and assessments. Participants are randomized to either the lifestyle intervention group or the control group following baseline screening. The intervention group receives 6 months of structured lifestyle support, including (1) dietary counseling, (2) individually tailored training with tutor support, (3) monthly group-based training, and (4) daily omega-3 and B-vitamin supplementation, while the control group receives TAU only. Data collection occurs at baseline, mid-intervention, and post-intervention (months 0, 3, and 6) in both arms. After the 6-month assessment, control group participants are offered the same lifestyle program as a delayed intervention, without further data collection. See Table 2 for outcome measures and Table 3 (SPIRIT schedule) for assessment timing.

Participants randomized to the intervention arm will receive a structured, multi-component lifestyle program in addition to TAU over a 6-month period. An overview of all intervention components, comparator conditions, and assessment schedule is provided in Figure 5, while Figures 3 and 4 illustrate the dietary component specifically. To ensure accessibility and continuity, all components of the intervention may be delivered in alternative venues if needed (e.g., during hospitalization). The intervention includes the following four core components (**2.3.1–4**).

Outdoor group sessions are held in a semi-rural setting near Sognsvann in Oslo, using NIH's facilities. Indoor alternatives will be offered during winter or poor weather. Transportation needs are addressed in collaboration with healthcare providers, such as FACT teams.

#### 2.3.1 Individually tailored training program

Each participant will receive a personalized training plan designed in collaboration with a physical activity professional from NIH. The program supports achieving ≥150 min of moderate-to-vigorous activity per week and will be adapted to the functional capacity, personal goals and preferences, as well as the resources and facilities each participant may have access to (e.g., gym access, home training, outdoor opportunities). To facilitate implementation, one individual coaching session will be conducted at baseline and another at mid-intervention (3 months). Participants will be encouraged to complete a short weekly training diary, sent by SMS with a link to Nettskjema (a secure online questionnaire platform from the University of Oslo), to monitor adherence. The clinical dietitian and training instructors will ask about completion of training goals at each session.

#### 2.3.2 Omega-3 and B-vitamin supplementation

The updated European guidelines for treatment of CVD recommends that individuals at high cardiometabolic risk consider supplementation with long-chain omega-3 polyunsaturated fatty acids (EPA and DHA) (44). All participants in the intervention group will therefore receive daily omega-3 supplements throughout the study period. The product (Möller's Trankapsler) contains a combined dose of 1,000 mg EPA/DHA per day (two capsules). Participants are instructed to take the capsules with a meal to enhance absorption and minimize gastrointestinal side effects. In addition, daily B-vitamin supplementation will be provided to support one-carbon metabolism and optimize the neuroprotective potential of omega-3 fatty acids, as suggested by previous studies demonstrating synergistic effects of B-vitamins and omega-3 fatty acids on cognitive outcomes (39). The product (Nycoplus B-Kompleks Ekstra Sterk) contains, per daily tablet, the following doses: thiamine (B1) 22 mg, riboflavin (B2) 22 mg, niacin 35 mg NE, vitamin B6 11 mg, folic acid 400 μg, vitamin B12 100 μg, pantothenic acid 45 mg, and biotin 150 μg.

#### 2.3.3 Tailoring to treatment context

The intervention will be adapted to each participant's treatment setting whether inpatient, outpatient, or community-based (e.g., FACT, a multidisciplinary outreach model providing flexible support to people with severe mental illness or substance abuse in their daily lives). Core program components remain consistent, while the delivery method is slightly adjusted to ensure feasibility, continuity, and accessibility. This flexibility also allows participants to continue study participation even if their treatment setting changes during the study period (e.g., changes from outpatient to inpatient, or vice versa). Importantly, the intervention is delivered as an adjunct to TAU, and participation in the study does not interfere with or replace any existing psychiatric care.

#### 2.3.4 Eligibility of intervention providers

Dietary counseling is delivered by registered clinical dietitians who are experienced in treatment of psychiatric patients. Physical activity sessions are led by certified instructors from NIH or staff within mental health services, such as physiotherapists trained in psychomotor therapy or exercise specialists with psychiatric expertise. All providers receive structured training in the intervention protocol to ensure consistent delivery across settings.

#### 2.3.5 Involvement of Significant Others (S.O.s)

To support long-term behavior change, participants in the intervention group are encouraged to involve a S.O., such as a family member, friend, or caregiver. Involvement is voluntary and tailored to the participant's preferences and needs.

S.O.s may be invited to attend individual consultations with the clinical dietitian, supervised physical activity sessions, or other follow-up appointments where appropriate. They will be offered relevant educational material to help foster a supportive home environment. Involving S.O.s may help address practical barriers and reinforce motivation by promoting a sense of shared responsibility and team spirit around the lifestyle changes undertaken. Initial insights from a feasibility study suggest that such involvement was well received and contributed positively to engagement and support for individuals with SMI (45). The presence and nature of S.O. involvement will be documented and considered in the process evaluation.

#### 2.3.6 Intervention fidelity

To ensure consistent delivery across participants and settings, fidelity procedures are implemented for all components of the intervention. All participants in the intervention arm receive the same frequency and structure of follow-up, regardless of motivation, engagement, or progress. All intervention activities follow the predefined protocol, and any deviations or amendments will be documented. The intervention is therefore standardized in dose, while allowing individual tailoring within predefined protocol boundaries.

Dietary component fidelity is ensured through the use of a fixed counseling framework [LEVA (43)], with standardized consultation and documentation routines. Each participant receives monthly consultations and biweekly follow-up calls, following identical procedural steps. Tailoring is limited to the choice of dietary step and behavioral goals, not to the intensity or frequency of follow-up. Training component fidelity is maintained by providing all participants with the same schedule of supervised monthly group sessions, and two counseling sessions with exercise professionals during the intervention period, during which a structured individual training plan is developed. Adherence to the individual training plan is monitored through a weekly digital training diary submitted via Nettskjema following SMS notification. The diary documents frequency, duration, mode of activity, and intensity of the previous week's training. Review of weekly goals takes place informally during group sessions and during consultations and follow-up calls with the clinical dietitian.

Supplement fidelity is maintained by dispensing one container of omega-3 and B-vitamin supplements at a time, with refills provided during dietary counseling sessions as needed. Compliance is assessed through follow-up calls, participant self-report, and confirmation of whether supplement containers have been used and require replacement at each consultation.

### 2.4 Control group

Participants allocated to the control group will continue receiving TAU, in line with their existing psychiatric care plans. TAU may include outpatient follow-up, pharmacological treatment, psychosocial support, and regular monitoring of somatic health parameters by the mental health service or general practitioner, depending on the participant's care setting. No restrictions will be placed on concomitant treatment or frequency of clinical contact in either arm.

To reduce contamination of the control setting, no lifestyle advice or counseling related to nutrition or physical activity will be provided during assessment visits during the study period. An overview of assessments during the control period is outlined in Figure 5. Randomization and disclosure of group allocation will take place only after baseline assessments are completed to further reduce possible contamination associated with assessments.

Following the final follow-up assessments, participants in the control group will be offered the same lifestyle program as a delayed intervention, with no further data collection. This ensures equitable access while maintaining the integrity of the primary outcome analysis.

### 2.5 Modification, discontinuation, and harms

Participants may withdraw from the study at any time without providing justification, as outlined in the PIS approved by the Norwegian Research Ethics Committee (Appendices). The intervention may be adapted to accommodate individual needs, functional capacity, or health status, in agreement with the study team and relevant clinical providers.

Although the trial poses minimal risk, any adverse events related to physical activity, dietary changes, or dietary supplementation will be documented and assessed. Participants who initiate or substantially modify pharmacological treatment listed as exclusion criteria in Table 1 during the intervention period will be excluded from per-protocol analyses but will remain in the intention-to-treat population unless they request withdrawal or meet predefined safety-related discontinuation criteria. Stable medication use at baseline does not affect eligibility. This strategy ensures that per-protocol analyses reflect the intended effects of the lifestyle intervention while maintaining data integrity in accordance with CONSORT guidelines (46). All participants will continue receiving usual psychiatric and medical care. Concomitant treatments and clinical changes will be documented at each assessment visit.

### 2.6 Outcome measures

Outcomes will be assessed at baseline, mid-intervention (3 months), and post-intervention (6 months), unless otherwise specified. The primary objective is to compare changes in CVD risk and related health outcomes between the intervention and control groups following the 6-month lifestyle program. All outcome measures are summarized in Table 2 and in the SPIRIT table (Table 3). All outcomes have been preregistered at clinicaltrials.gov [NCT07085923] prior to study start.

**Table 2:** Data collected in the study.

**Data category** **Measurement method**	**Anthropometry** **Physical measurements and BIA**	**Biochemistry** **Blood biomarkers**	**Dietary intake** **Questionnaire and interview**	**PROM** **Questionnaire**	**CVD-risk** **Risk prediction algorithm**
Variables	Body weight Height BMI Waist circumference Blood pressure Heart rate Body composition measured by BIA	**Lipid profile** Total C LDL-C HDL-C Triglycerides ApoB Lp(a) **Glucose markers:** Fasting glucose HbA1c **Vitamins and methylation marker**^**a**^**:** Folate Vitamin D Vitamin B12 Homocystein^a^ **Liver enzymes:** ALT AST Homocysteine **Thyrroid hormones:** TSH T4 **Inflammatory marker:** Hs-CRP **Neurocognitive markers (biobank):** NfL Ptau-217	24-hour dietary recall interview DIGIKOST	PROMIS-29	QRISK3-2018
			**Physical activity**	**Qualitative data**	
			Questionnaire and accelerometer	Field notes	
			Actigraph- accelerometer DIGIKOST	Relevant observations and citations	

Frequency of data collection is outlined in the SPIRIT-form (Table 3).

**BIA**, Bioelectrical Impedance Analysis; **BMI**, Body Mass Index; **ALT**, Alanine Aminotransferase; AST, aspartate aminotransferase; **C**, Cholesterol; **LDL-C**, Low-Density Lipoprotein Cholesterol; **HDL-C**, High-Density Lipoprotein Cholesterol; **ApoB**, Apolipoprotein B; **Lp(a)**, Lipoprotein(a); **HbA1c**, Glycated Hemoglobin; **hs-CRP**, High-Sensitivity C-Reactive Protein; **TSH**, Thyroid-Stimulating Hormone; **T4**, Thyroxine; **NfL**, Neurofilament Light chain; **ptau-217**, phosphorylated tau protein-217; **PROM**, Patient Reported Outcome Measures.

**Table 3:** SPIRIT figure schedule of enrolment, interventions and assessments.

**Time point**	Study period									
	**Enrolment w0/month0**	**Allocation w0**	**w1–2**	**w1–4**	**w5–8**	**Mid Intervention w 9–12/month3**	**w13–16**	**w17–20**	**w21–24**	**Post Intervention w24–25/month6**
**Enrolment**										
Eligibility screen	x									
Informed consent	x									
Baseline data	x					x				x
Journal information			x			x				x
Allocation		x								
**Interventions**										
Dietary counseling			x		x	x	x	x	x	
Group training				x	x	x	x	x	x	
Training instruction			x			x				
n3 supplementation			x	x	x	x	x	x	x	
**Assessments**										
Journal Information	x					x				x
Anthropometry	x				x	x	x	x	x	x
Body Composition	x				x	x	x	x	x	x
Height	x									
Blood samples	x					x				x
Dietary assessment^a^	x									x
Physical activity^b^	x									x
QOL(PROMIS-29)	x					x				x
CVD-RISK	x					x				x
Field notes	x	x	x	x	x	x	x	x	x	x

Data collected in the study are specified in Table 2. Figure 3 visualizes study activities following randomization in both arms.

^a^DIGIKOST-questionnaire and 24 h recall-interviews, ^b^DIGIKOST-questionnaire and objectively measured physical activity using accelerometer.

N3, omega-3 fatty acids; QoL, Quality of Life; PROMIS-29, Patient Reported Outcome Measure Information System 29-item version; CVD, Cardiovascular Disease.

#### 2.6.1 Primary outcomes

- **Change in estimated 10-year CVD risk** Assessed as the between-group difference in QRISK3 score from baseline to 6 months, using the QRISK3 algorithm (47).
- **Association between change in lifestyle adherence and change in QRISK3 score** Evaluated using the DIGIKOST Lifestyle Index (40) at baseline and 6 months, with statistical models assessing the relationship between changes in adherence to a healthy lifestyle and changes in QRISK3 scores.

#### 2.6.2 Secondary outcomes

- **Change in objectively measured physical activity**.

Assessed using a waist-worn accelerometer, worn for seven consecutive days at baseline and 6 months.

- **Change in lifestyle adherence score**.

Measured by the DIGIKOST Lifestyle Index from baseline to 6 months.

- **Change in dietary intake**.

Evaluated using the DIGIKOST Diet Scores and repeated 24-hour dietary recalls conducted at baseline and 6 months.

- **Presence and change in metabolic syndrome**.

Defined according to International Diabetes Federation and Adult Treatment Panel III criteria (48), assessed at baseline, 3 months, and 6 months.

- **Change in nutrition-related blood biomarkers**

Includes total cholesterol, low-density lipoprotein cholesterol (LDL-C), high-density lipoprotein cholesterol (HDL-C), triglycerides, apolipoprotein B (ApoB), lipoprotein(a) [Lp(a)], glucose, glycated hemoglobin (HbA1c), alanine aminotransferase (ALT), aspartate aminotransferase (AST), folate, 25-hydroxyvitamin D, homocysteine, high-sensitivity C-reactive protein (hs-CRP), thyroid-stimulating hormone (TSH), and thyroxine (T4). Changes will be assessed from baseline to three and 6 months, comparing the intervention group to the control group.

- **Change in body weight**.

Measured at baseline, 3 months, and 6 months in both groups using calibrated digital scales. In the intervention group, this measure will also be recorded during monthly consultations to monitor individual progress and support personalized feedback.

- **Change in waist circumference**.

Measured in duplicate at baseline, 3 months, and 6 months in both groups using a non-stretch tape measure. In the intervention group, this measure will also be recorded during monthly consultations to monitor individual progress and support personalized feedback.

- **Change in blood pressure**.

Systolic and diastolic pressure assessed using validated automated devices at baseline, 3 months, and 6 months in both groups.

- **Change in body composition**.

Assessed by bioelectrical impedance analysis (BIA) at baseline, 3 months, and 6 months in both groups. In the intervention group, this measure will also be recorded during monthly consultations to monitor individual progress and support personalized feedback.

#### 2.6.3 Exploratory outcomes

- **Change in health-related quality of life**.

Assessed using the Patient-Reported Outcomes Measurement Information System (PROMIS)-29 item questionnaire at baseline, 3 months, and 6 months in both groups. This questionnaire measures health-related quality of life, covering seven domains: physical functioning, anxiety, depression, fatigue, sleep disturbance, social participation, and pain interference, as well as a global quality of life-score. It has been validated for use in both general and clinical populations, including individuals with mental health conditions (49).

- **Feasibility and acceptability of the lifestyle programme**.

Evaluated through qualitative field notes recorded by the clinical dietitian and physical activity instructors throughout the study period. These may include informal feedback and participant quotations from both study arms, but without structured interview guides.

- **Predictors of adherence and differential intervention effects**.

Analyses will explore whether baseline variables (e.g., diagnosis, sex, BMI, medication use) are associated with intervention adherence or act as effect modifiers.

- **Biomarkers of neurocognitive decline**.

Plasma concentrations of NfL and p-tau217 will be analyzed to explore associations between cardiometabolic and neurocognitive vulnerability throughout the study period.

#### 2.6.4 Process evaluation: degree of S.O.-involvement and use of GBO

In the intervention group, involvement of S.O.s will be documented as part of the process evaluation. This includes a binary yes/no variable indicating whether the participant chose to involve an S.O., and the number of sessions (e.g., dietary consultations or training sessions) attended by the S.O. This information will be used to describe the extent of S.O. involvement and may inform exploratory analyses of supportive structures. In addition, GBO, used to capture participants' self-defined goals and perceived progress during dietary counseling sessions, will provide insight into their experiences with the intervention and may inform its future clinical application.

### 2.7 Data collection and management

Data are primarily collected at three scheduled time points: baseline, mid-intervention (3 months), and post-intervention (6 months). The full assessment schedule is presented in the SPIRIT table (50) (Table 3) and visualized relative to group allocation in Figures 2 and 5. An overview of variable domains and data sources is provided in Table 2.

#### 2.7.1 Dietary assessment

Dietary habits are assessed using the DIGIKOST food-frequency questionnaire and two non-consecutive 24-h dietary recall interviews, conducted first in person and then repeated by phone a few days later. Both dietary assessments are performed at baseline and 6 months to reduce participant burden and avoid potential contamination in the control group. The DIGIKOST tool has been validated for use in Norwegian populations and reflects adherence to national dietary recommendations (40). This questionnaire will be completed digitally via Nettskjema in the presence of the clinical dietitian, who will be available to clarify any questions. The 24-h recalls will be administered by the clinical dietitian, first in person and subsequently by telephone. Visual aids, including standardized food images and portion guides, will be used as needed to improve recall accuracy. Nutrient intake will be calculated as the mean of the two non-consecutive 24-h recalls and analyzed using the validated Norwegian food composition database in NutriFoodCalc (NFC, v1.0).

#### 2.7.2 Physical activity

Objectively measured physical activity is assessed using a waist-worn accelerometer (ActiGraph wGT3X-BT), worn continuously for seven consecutive days at both baseline and 6 months. The device captures posture and movement patterns, including sedentary time, standing, walking, and moderate-to-vigorous physical activity. In addition, self-reported data on physical activity and sedentary behavior are collected through the DIGIKOST questionnaire. Combining these sources provides a broader understanding of activity patterns and enables cross-validation between objective and self-reported measures.

#### 2.7.3 Anthropometry, blood pressure, and heart rate

Body weight, waist circumference, and blood pressure are measured in both groups at baseline, 3 months, and 6 months. In the intervention group, these assessments are also conducted at each monthly visit with the clinical dietitian to support progress monitoring and participant engagement. Participants are further advised to monitor their body weight at home weekly under standardized routines, which is followed up during biweekly phone calls. Bathroom scales are available for loan if needed.

**Body weight** is measured using a calibrated digital scale, with participants wearing light clothing or underwear and no shoes. Toilet visits are encouraged prior to weighing, and measurements are ideally taken in a fasted state. **Waist circumference** is measured in duplicate at the midpoint between the lowest rib and iliac crest using a non-elastic measuring tape, recorded at the end of a normal exhalation. **Blood pressure** is assessed on the right arm using a validated automatic device following at least 10 min of seated rest. Three consecutive readings will be obtained, and the mean values of systolic and diastolic blood pressure will be calculated. Resting heart rate will be recorded during the first blood pressure measurement. Participants are instructed to avoid alcohol, sauna use, and vigorous physical activity for at least 24 hours prior to measurement, and to be fasted (including coffee or other caffeine-containing beverages) for at least 8 h before data assessment.

#### 2.7.4 Body composition

Body composition, including total body fat percentage and skeletal muscle mass, is assessed using multifrequency bioelectrical impedance analysis (BIA) with the seca mBCA [554] (Seca GmbH & Co. KG, Hamburg, Germany). Measurements are conducted at baseline, 3 months, and 6 months in both groups, with monthly tracking in the intervention group. The same pre-assessment instructions apply as for anthropometry.

#### 2.7.5 Blood samples

Fasting blood samples are drawn at baseline, 3 months, and 6 months by the project investigator or trained bioengineers. Participants are instructed to fast for a minimum of 8 h and avoid the beforementioned confounding exposures before sampling. Biomarkers include a lipid profile (total cholesterol, LDL-C, HDL-C, triglycerides, ApoB, ApoA1, Lp(a)), glycaemic markers (glucose, HbA1c), hepatic enzymes (ALT, AST), vitamins and one-carbon metabolism (vitamin D, B9, B12, and homocysteine), inflammatory marker (hs-CRP), and thyroid hormones (TSH, T4), as outlined in Table 2. Laboratory procedures are conducted by the project investigator at Domus Medica, and all analyses are performed at Fürst Medical Laboratory in Oslo.

##### 2.7.5.1 Blood samples stored in biobank

An additional plasma sample is collected from each participant at every visit and stored at −80 °C in a project-specific biobank at the University of Oslo. These samples will be used for later analyses of neurocognitive biomarkers, including p-tau217 and NfL. Analyses will be conducted in collaboration with Sahlgrenska University Hospital in Gothenburg.

#### 2.7.6 Health-related quality of life

The PROMIS-29 questionnaire is used to assess health-related quality of life at all three main visits. It will be completed digitally via Nettskjema. Raw domain scores will be converted to T-scores (mean 50, SD 10) using standard scoring manuals.

#### 2.7.7 Qualitative data

Field notes are recorded throughout the study period in both groups by the clinical dietitian and exercise instructors. These may include participant quotations, informal feedback, and contextual observations gathered during consultations, training sessions, or telephone contact. No structured interview guide is used, and data collection is embedded within routine interactions.

This unstructured, opportunistic approach has previously proven valuable for capturing participants' own reflections on their physical health, lifestyle habits, and experience of lifestyle guidance (19). Such observations may provide important nuance to the quantitative data and offer insight into perceived barriers, facilitators, and motivation for change.

#### 2.7.8 Data entry, storage, and confidentiality

All data management procedures adhere to the General Data Protection Regulation and relevant Norwegian data protection legislation (51). To ensure confidentiality, each participant is assigned a unique study ID generated at the time of enrolment. This ID is used for all data collection, storage, and analysis. A separate enrolment log links participant names and personal identification numbers to their study ID, and this file is stored independently from the research dataset.

Research data, including questionnaire responses, biometric measurements, and qualitative field notes, are stored on the Services for Sensitive Data (TSD) platform at the University of Oslo, which provides a secure infrastructure for data entry, processing, and long-term storage. Journal notes taken during clinical consultations with the clinical dietitian are also recorded and stored in TSD. Sikt, the Norwegian Agency for Shared Services in Education and Research, serves as the official data protection officer and data processor for the project (reference: 108677), ensuring institutional and legal compliance.

Access to data is restricted to authorized study personnel using two-factor authentication, and all access is logged. The study team is responsible for monitoring data quality throughout the study period, following predefined procedures for identifying and resolving missing data, inconsistencies, or outliers. No external audit is planned due to the investigator-initiated nature and limited scale of the trial, but data handling will follow the protocols developed in collaboration with and approved by Sikt.

No personal identifiers will be used in analysis or publications. Research data will be stored for a minimum of 5 years after study completion, in accordance with institutional policy, after which secure deletion will be carried out unless extended retention is required.

### 2.8 Statistical analysis

#### 2.8.1 Statistical power and sample size

The sample size was determined based on the primary outcome: change in estimated 10-year CVD- risk using the QRISK3 algorithm (26). QRISK3 was selected due to its inclusion of SMI and antipsychotic medication use as risk factors, its suitability for adults from age 25, and its broader predictive performance compared to other commonly used tools such as Framingham and PRIMROSE (52).

Lifestyle interventions in Nordic populations without SMI have shown relative CVD-risk reductions of 14–22% (27, 28). In this trial, a 20% relative reduction in QRISK3 score is considered a realistic and clinically meaningful effect size, corresponding to an absolute reduction of approximately 3–6% in estimated 10-year CVD risk. Drawing on observed variability in multivariable CVD-risk estimates reported in lifestyle intervention studies (27, 28), the standard deviation for the expected change in risk was conservatively set at 7 percentage points. Assuming a baseline QRISK3 risk score of 30%, a total of 46 participants (23 per group) would provide 80% power to detect a 20% relative reduction at a two-sided 5% significance level. This is considered plausible based on available evidence showing markedly elevated CVD risk in individuals with SMI (53). Allowing for up to 30% attrition, the target sample size was set to 70 participants (35 per group). This estimate is considered conservative; a smaller trial in individuals with SMI reported a 21% dropout rate during a 6-week dietary intervention (21).

#### 2.8.2 Primary and secondary outcome analyses

Baseline characteristics of the study sample will be presented by usual descriptive measures. The study groups will be presented separately, based on the intention-to-treat principle. The initial analysis will be performed based on all participants who provided baseline data. The primary outcome, change in estimated 10-year CVD risk, will be analyzed by Analysis of Covariance (ANCOVA) with the estimated risk at baseline as covariate. The other primary outcome, association between change in lifestyle adherence and change in QRISK3 score, will be investigated using a simple linear regression model. Similar analyses (ANCOVA) will be conducted for secondary outcomes measured on a continuous scale, at baseline and after 6 months (physical activity, lifestyle adherence score, dietary intake). Continuous secondary outcomes measured more frequently (at baseline, after 3 and 6 months) will be analyzed by a linear mixed model with random intercept and random effect of time and fixed effects of group, time and group by time interaction (biomarkers, body weight, waist circumference, blood pressure). The last secondary outcome, presence of metabolic syndrome, will be analyzed by a logistic mixed model in the same way as above.

#### 2.8.3 Missing data handling

Missing data relevant for the analysis of primary or secondary outcomes will be imputed using multiple imputation, assuming data are missing at random (MAR).

#### 2.8.4 Sensitivity analyses

Sensitivity analyses will be conducted to assess the robustness of the results to assumptions regarding missing data and potential model violations. These analyses will examine the influence of outliers, alternative missing-data mechanisms (including deviations from the MAR assumption), and participant dropout. Furthermore, all primary and secondary outcomes will be analyzed with an adjustment for potential confounders to investigate the influence of imbalance in the randomized groups.

## 3 Discussion

The NORMI-Heart study aims to contribute evidence to the prevention of CVD in individuals with SMI, a population characterized by persistently elevated cardiometabolic risk and limited access to tailored lifestyle support. By implementing a structured, yet flexible, lifestyle program in real-world psychiatric care, the study aims to evaluate both effectiveness and feasibility across treatment settings for individuals with SMI in Norway. The study is investigator-initiated and integrated into existing psychiatric care pathways. It has been designed to ensure feasibility and acceptability for both participants and healthcare providers without disrupting routine clinical practice.

### 3.1 Strengths and limitations

Key strengths include the pragmatic design and delivery by professionals with relevant experience. The dietary intervention is led by a clinical dietitian (35) with experience from psychiatric care, ensuring evidence-based dietary guidance tailored to individuals with SMI. The methodology in the dietary counseling component draws on a Scandinavian trial that demonstrated long-term improvements in diet quality and energy intake using a rigorous and structured approach to calorie reduction and weight loss (43). In the NORMI-Heart trial, this framework has been developed in collaboration with clinical dietitians and a user advisory panel to ensure clinical relevance and acceptability in the SMI population. Core strategies for caloric reduction are retained, but major methodological modifications have been introduced: a weighed food record was considered impractical and too comprehensive, and dietary assessment will instead rely on a Norwegian-adapted FFQ and repeated 24 h recall interviews. Participants will also be encouraged to prioritize dietary goals themselves, in line with the GBO framework and clinical best practice in dietary counseling. While this flexible approach is expected to improve feasibility and engagement, it may also introduce greater variability in implementation. Although GBO data are not part of the primary outcome analysis, they are expected to inform the evaluation of feasibility and adherence and may provide clinically useful insights for future implementation.

The physical activity component emphasizes sufficient moderate-to-vigorous intensity physical activity, in line with current evidence (31). Importantly, the monthly group-based training sessions are intended primarily as a motivational tool, fostering belonging, social support, and inspiration, rather than to provide the full recommended activity dose. Some clinicians have suggested that group training may be poorly received by SMI patients with social anxiety; however, input from the user panel indicated that group activity can strengthen adherence. The optional involvement of S.O.s may enhance social support and compliance to both dietary- and exercise goals (45). Provision of omega-3 supplementation may contribute to reducing cardiometabolic risk markers in our trial, particularly in those with a suboptimal fish intake, which is common in Norway (54). Each intervention component is delivered by professionals with relevant expertise, and the program was developed through multidisciplinary collaboration, expected to support fidelity, engagement, and scalability.

Eligibility criteria were defined to include individuals at high cardiometabolic risk, thereby enhancing clinical relevance, while exclusion criteria were designed to reduce clinical risk and avoid confounding, supporting both participant safety and internal validity. Repeated assessment of both physiological and self-reported outcomes strengthens the evaluation by capturing changes in cardiometabolic and mental health parameters over time, and qualitative data from unstructured field notes may further contextualize participants' experiences with the intervention.

Some limitations should be acknowledged. The feasibility and degree of S.O. involvement may vary between participants. Differences in care context, including treatment intensity and follow-up routines, could also influence outcomes. Furthermore, the Oslo-based clinical setting may limit generalizability to regions with more constrained resources or less integrated psychiatric services. The frequency of supervised training sessions and dietary follow-up is limited by available resources, which may reduce the intensity of support that is needed to achieve optimal adherence in SMI patients. Moreover, the study is not powered to disentangle the specific contributions of diet vs. physical activity. However, the intervention is grounded in national guideline-based recommendations for individuals with SMI (24), where both diet and physical activity are prioritized (17). Integrating these components enables evaluation of the combined impact of behavioral and nutritional strategies on cardiometabolic vulnerability in SMI. The open-label design introduces a risk of performance and detection bias, although blinded baseline assessments and objective primary outcomes help mitigate these effects.

If successful, the NORMI-Heart study may provide a foundation for broader implementation of cardioprotective interventions in psychiatric care, including justification for increased availability of clinical dietitians across services. Importantly, this may be the first trial to test the feasibility and clinical utility of a structured dietary intervention in individuals with SMI, combined with a patient-centered approach where participants define their own goals. Such knowledge could help shape future practice and inform scalable models of lifestyle support in mental health services. Moreover, exploratory analyses of neurocognitive biomarkers may yield novel insight into the shared mechanisms underlying cardiovascular and dementia risk in SMI, providing a unique contribution to an underexplored field.

## 4 Ethics and user involvement

### 4.1 Ethical approval

The study protocol has been approved by the Regional Committees for Medical and Health Research Ethics in Norway (reference: 865976, approved on [04.06.2025]). All participants will receive a PIS and provide written informed consent prior to inclusion (Appendices).

### 4.2 User involvement

User involvement has been integrated throughout the development and planning of the study. A multidisciplinary user panel has contributed insights from diverse perspectives, including individuals with lived experience of SMI and S.O.'s, peer-support specialists, and health professionals working in psychiatric care. Ongoing collaboration with the panel is planned through regular meetings to support process evaluation, interpretation of findings, and clinical application. This approach aims to ensure meaningful and continuous involvement, rather than treating user engagement as a complementary “add-on” in the research process.

## 5 Trial registration and status

The study is registered at ClinicalTrials.gov with the identifier NCT07085923, submitted prior to participant enrolment. Recruitment began on 16 September 2025. At the time of resubmission, 15 participants (21.4% of the planned sample) have been recruited and randomized. Recruitment will continue until the target sample size has been reached. Any significant changes to the protocol, study design, or outcome measures will be updated in the trial registry to ensure transparent research process and submitted to Regional Committees for Medical and Health Research Ethics for approval prior to implementation.

## 6 Dissemination and advertisement plan

Recruitment is expected to be ongoing until January 2027 to ensure sufficient participant enrolment and statistical power to address the predefined study objectives. The study will be promoted continuously within the participating clinical sites and through collaboration with relevant user organizations. In addition, recruitment advertisements will be shared in local newspapers and on social media platforms using accessible and inclusive language. A study-specific website with information and contact details has also been published on the Institute of Basic Medical Sciences, University of Oslo website: https://www.med.uio.no/imb/forskning/prosjekter/normi/index.html.

Findings from the study will be disseminated through peer-reviewed scientific publications and conference presentations targeting both psychiatric and public health audiences. Results will also be communicated to healthcare providers, mental health services, user organizations, and relevant professional networks. A plain-language summary will be provided to all participants, and educational materials may be developed to support the clinical implementation of heart-healthy lifestyle interventions in psychiatric care. The user panel will contribute actively to the planning and execution of the dissemination strategy to ensure relevance, accessibility, and meaningful communication of study outcomes.
