# Correction: Clinical dietitian-led nutrition counseling and exercise to reduce cardiovascular risk in adults living with a BMI above 27 and severe mental illness: the NORMI-Heart trial protocol

**DOI:** 10.3389/fnut.2026.1812726

**Published:** 2026-03-11

**Authors:** Madeleine Elisabeth Angelsen, Hemen Najar, Mette Svendsen, Magne Thoresen, Dawn Elizabeth Peleikis, Kjetil Retterstøl

**Affiliations:** 1 Department of Nutrition, Faculty of Medicine, Institute of Basic Medical Sciences, University of Oslo, Oslo, Norway; 2 Department of Psychotic Disorders, Sahlgrenska University Hospital, Västra Götaland Region, Gothenburg, Sweden; 3 Institute of Health and Care Sciences, University of Gothenburg, Gothenburg, Sweden; 4 Section for Preventive Cardiology, Department for Endocrinology, Obesity and Preventive Medicine, Oslo University Hospital, Oslo, Norway; 5 Department of Biostatistics, Faculty of Medicine, Institute of Basic Medical Sciences, University of Oslo, Oslo, Norway; 6 District Psychiatric Center (DPS) of Asker, Vestre Viken Hospital Trust, Drammen, Norway; 7 The Lipid Clinic, Medical Department, Aker Hospital (Aker Sykehus), Oslo University Hospital (Oslo universitetssykehus), Oslo, Norway

**Keywords:** cardiovascular disease, clinical dietitian, heart health, lifestyle intervention, metabolic risk factor, psychiatry, severe mental illness, weight reduction

Affiliation “Section for Preventive Cardiology, Department for Endocrinology, Obesity and Preventive Medicine, Oslo University Hospital, Oslo, Norway” was omitted from the published article. This affiliation has now been added as affiliation 4.

In addition, author Mette Svendsen was erroneously assigned to affiliations 2 and 3 “Department of Psychotic Disorders, Sahlgrenska University Hospital, Västra Götaland Region, Gothenburg, Sweden”; and “Institute of Health and Care Sciences, University of Gothenburg, Gothenburg, Sweden.”

The correct affiliations for Mette Svendsen are 4 “Section for Preventive Cardiology, Department for Endocrinology, Obesity and Preventive Medicine, Oslo University Hospital, Oslo, Norway” and 1 “Department of Nutrition, Faculty of Medicine, Institute of Basic Medical Sciences, University of Oslo, Oslo, Norway”).

The original version of this article has been updated.

