# Supplementary material for: Clinical dietitian-led nutrition counseling and exercise to reduce cardiovascular risk in adults living with a BMI above 27 and severe mental illness: the NORMI-Heart trial protocol
Source: Front Nutr. 2026 Feb 12;13:1700251. doi: 10.3389/fnut.2026.1700251 (PMC12935603; doi:10.3389/fnut.2026.1700251)
Supplement: Supplementary file 1 [file Supplementary_file_1.pdf]

CAN A HEALTHY LIFESTYLE REDUCE THE RISK OF CARDIOVASCULAR DISEASE IN PEOPLE WITH SEVERE MENTAL ILLNESS?

PURPOSE OF THE PROJECT AND WHY YOU ARE INVITED

You are being invited to take part in this research project because you are currently receiving treatment for a severe mental illness. Research shows that people with severe mental illness have a higher risk of cardiovascular disease, partly due to lifestyle factors and side effects of medication. The purpose of this study is to investigate whether a structured lifestyle programme—including dietary counselling, physical activity, and supplementation with selected nutrients (omega-3 and B-vitamins)—can affect mental and physical health in this patient group, including markers of brain function and heart health.

WHAT DOES PARTICIPATION INVOLVE?

Participation involves an assessment of your health and lifestyle, after which you will be randomly assigned to either an intervention group receiving lifestyle guidance or a control group receiving standard care for the first six months. After this period, the control group will also be offered the same lifestyle programme, ensuring that all participants receive an equivalent health-promoting intervention.

Participation includes:

1. Health assessment:

Ved At baseline, we will measure your weight, height, waist circumference, blood pressure, body composition (using BIA), and physical activity (using an accelerometer), in addition to taking blood samples. One blood sample will also be stored in a biobank. You will complete questionnaires about diet, physical activity, and mental health. Information will be collected from your medical record or responsible clinician regarding diagnosis, medication use, previous weight and medical history, and family history of cardiovascular disease. These assessments will be repeated after three months and at the end of the intervention period.
After the baseline assessment, you will be randomly assigned to the intervention group (A) or the control group (B).

2A) Intervention Group:

If you are allocated to the intervention group, you will participate in a six-month lifestyle programme in addition to your usual care. The goal is to support you in eating more healthily, becoming more physically active, and losing weight if you wish. You will meet with a clinical dietitian once a month (six sessions in total) for dietary counselling and follow-up. At each meeting, your weight, blood pressure, waist circumference and body composition will be measured. You will keep a food diary during parts of the intervention. You will receive an exercise programme to follow twice weekly, and you will have two individual follow-up sessions with an exercise instructor during the six-month period. You will also be invited to one group exercise session each month (six sessions in total). The programme is adapted to your needs, and you will receive support to get started. In addition, you will receive dietary supplements (fish-oil capsules/omega-3) and a B-vitamin supplement to take daily throughout the intervention period. The purpose is to examine how supplementation combined with lifestyle guidance influences biological markers. After three months (midway through the study), the same assessments as at baseline will be repeated.

2B) Control Group:

If you are allocated to the control group, you will continue with your usual care for six months. Halfway through the study (three months after baseline) and at the end of the study (after six months), you will be

asked to attend the same assessments as at baseline. Afterwards, you will be offered the same lifestyle programme as the intervention group. No new measurements will be taken during this later period.

3. **Final assessment:**

The same assessments as at baseline will be repeated after six months to evaluate any changes.

By participating, you consent to your responsible clinician at Oslo University Hospital / Lovisenberg Diaconal Hospital / Diakonhjemmet Hospital, or your GP, being contacted if you experience any unexpected serious psychological or physical reactions during the study that require medical or psychological follow-up — although such events are not expected.

POSSIBLE BENEFITS AND RISKS

Taking part in the study may increase your insight into your own health and lifestyle and gives you access to individual follow-up on diet and physical activity from qualified professionals. All participants will be offered the lifestyle programme, either immediately or after six months. However, we cannot guarantee health improvements. Lifestyle changes require personal effort, and it may be challenging to exercise regularly or change dietary habits.

Blood sampling may cause mild and temporary discomfort. You will receive dietary supplements (fish-oil capsules and B-vitamins), and no side effects are expected at the recommended doses. You will also be asked to complete questionnaires about your mental health and discuss your diet and lifestyle, which some people may find sensitive or emotionally demanding. If anything discussed during the study feels difficult, your project contact can, if you wish, relay this to your clinician for further follow-up.

VOLUNTARY PARTICIPATION AND WITHDRAWAL

Participation in the project is voluntary. If you wish to participate, you sign the consent form on the last page. You may withdraw your consent at any time without giving a reason. This will not have any negative consequences for you or your treatment. If you withdraw, your data and biological material will no longer be used for research. You may request access to the information stored about you, which will be provided within 30 days. You may also request that your data be deleted and that the biological material be destroyed. The right to request deletion or destruction does not apply if the data or biological material have been anonymised or published. This right may also be limited if your data have already been included in completed analyses or if the material has been processed and incorporated into another biological product.
If you later wish to withdraw or have questions about the study, you may contact the project leader (see contact information on the last page).).

HOW YOUR INFORMATION WILL BE USED?

The information collected about you will only be used for the purposes described and is planned to be used until 2030, ensuring that important findings can be completed and communicated to relevant professionals and decision-makers. Any extension of use or storage requires approval from the Regional Committee for Medical and Health Research Ethics (REK) and other relevant authorities. The extra blood sample will be stored in a local research biobank at the Faculty of Medicine, University of Oslo, for later analyses related to the study aims.
You have the right to access your stored information and to correct any errors. You also have the right to information about security measures related to data processing. You may file a complaint with the Norwegian Data Protection Authority or the institution’s Data Protection Officer.

All information will be processed without your name, birth number, or other directly identifying details (= coded data). A code links you to your information through a key list, which only the project leader and relevant project staff can access. Everyone involved in the project is bound by confidentiality and will not share information about you without your consent.

After the project has ended, your information will be stored for five years for control purposes. If your information is stored outside the institution, this will be specified.

WHAT HAPPENS TO YOUR BLOOD SAMPLES?
Blood samples will be analysed for cardiovascular risk factors such as cholesterol, blood glucose and inflammatory markers. One additional tube of blood will be collected and securely stored at the University of Oslo for possible future analyses to improve understanding of how lifestyle changes influence heart and brain health. The samples will only be used for this project and will be destroyed no later than 31 December 2030.

LEGAL BASIS FOR PROCESSING YOUR DATA
Your data are processed for scientific research purposes and because the project is considered to be in the public interest. On behalf of the University of Oslo, Sikt – The Norwegian Agency for Shared Services in Education and Research – has assessed that the processing of personal data in this project complies with data protection regulations.

YOUR RIGHTS
As long as you can be identified in the data material, you have the right to object, access your data, and request correction or deletion. You will receive a response within one month. We will provide a justification if we believe that you cannot be identified or that your rights cannot be exercised. You also have the right to complain to the Norwegian Data Protection Authority about how your data are processed.

RESPONSIBLE INSTITUTIONS

The study is conducted by the University of Oslo (UiO) in collaboration with the Norwegian School of Sport Sciences, Diakonhjemmet Hospital, Oslo University Hospital, and Lovisenberg Diaconal Hospital. Professor Kjetil Retterstøl is the project leader at UiO, and PhD candidate Madeleine Angelsen is responsible for the practical conduct of the study and participant contact. Your treatment provider will continue to be responsible for your usual medical and psychiatric follow-up during the study. The fish-oil capsules used in the study are provided by Orkla Health AS (Möller's), and the B-vitamin supplement by Orifarm AS (Nycoplus). The companies have no financial interests in the project and no influence over how the study is conducted or how the results are published.

GODKJENNINGER

The Regional Committee for Medical and Health Research Ethics (REK) approved the project on 04.06.25.
Reference number: 865976.
UiO, represented by project leader Professor Kjetil Retterstøl and PhD candidate Madeleine Angelsen, is responsible for safeguarding data protection in the project. Data are processed because the project is considered to be in the public interest.

CONTACT INFORMATION

If you have questions about the project, you may contact:

Kjetil Retterstøl
Project Leader, Professor (Department of Nutrition, UiO) and Senior Consultant (Lipid Clinic, Oslo University Hospital)
E-mail: Kjetil.retterstol@medisin.uio.no
Phone:
Madeleine Angelsen
Ph.D. candidate and clinical dietitian
E-mail: m.e.angelsen@studmed.uio.no
Phone:

CONSENT TO PARTICIPATE AND TO THE USE OF MY PERSONAL DATA AND BIOLOGICAL MATERIAL AS DESCRIBED

Place and date   Participant's signature:

Participant's name in block letters:

I confirm that I have provided information about the project.

Place and date

Signature

Role in the project
